# Calhex231 ameliorates myocardial fibrosis post myocardial infarction in rats through the autophagy‐NLRP3 inflammasome pathway in macrophages

**DOI:** 10.1111/jcmm.15969

**Published:** 2020-10-12

**Authors:** Wenxiu Liu, Jiaxing Sun, Yutong Guo, Na Liu, Xue Ding, Xin Zhang, Jinyu Chi, Ningning Kang, Yue Liu, Xinhua Yin

**Affiliations:** ^1^ Department of Cardiology First Affiliated Hospital of Harbin Medical University Harbin China

**Keywords:** autophagy, calcium‐sensing receptor, macrophage, myocardial infarction, NLRP3 inflammasome

## Abstract

The calcium‐sensing receptor (CaSR) is involved in the pathophysiology of many cardiovascular diseases, including myocardial infarction (MI) and hypertension. The role of Calhex231, a specific inhibitor of CaSR, in myocardial fibrosis following MI is still unclear. Using Wistar rats, we investigated whether Calhex231 ameliorates myocardial fibrosis through the autophagy‐NLRP3 inflammasome pathway in macrophages post myocardial infarction (MI). The rats were randomly divided into sham, MI and MI + Calhex231 groups. Compared with the sham rats, the MI rats consistently developed severe cardiac function, myocardial fibrosis and infiltration of inflammatory cells including macrophages. Moreover, inflammatory pathway including activation of NLRP3 inflammasome, IL‐1β and autophagy was significantly up‐regulated in myocardial tissue, infiltrated cardiac macrophages and peritoneal macrophages of the MI rats. These impacts were reversed by Calhex231. In vitro, studies revealed that calindol and rapamycin exacerbated MI‐induced autophagy and NLRP3 inflammasome activation in peritoneal macrophages. Calhex231 and 3‐Methyladenine (a specific inhibitor of autophagy) attenuated both autophagy and NLRP3 inflammasome activation; however, the caspase‐1 inhibitor Z‐YVAD‐FMK did not. Our study indicated that Calhex231 improved cardiac function and ameliorated myocardial fibrosis post MI, likely via the inhibition of autophagy‐mediated NLRP3 inflammasome activation; this provides a new therapeutic target for ventricular remodelling‐related cardiovascular diseases.

## 1 INTRODUCTION

Myocardial infarction (MI), a serious cardiovascular event, is the main cause of cardiac death.^1^ The inflammatory response is critical in cardiac healing after MI; however, excessive inflammation can lead to adverse pathological remodelling such as cardiac enlargement, myocardial fibrosis and then cardiac dysfunction.^2^ Macrophages, as the main response cells after MI, participate in the regulation of wound healing post MI,^3^ in several ways including clearing necrotic cardiomyocytes, secreting cytokines such as interleukin 1 beta (IL‐1β) and chemokines.^4^ IL‐1β promotes ventricular remodelling by inducing myocardial inflammation following MI.^5^, ^6^ The NLRP3 inflammasome promotes the activation and secretion of inflammatory cytokines IL‐1β and IL‐18,^7^ and contributes to inflammation and infarct size post MI.^8^

Autophagy is a conservative process of intracellular component recovery and degradation.^9^ Studies have shown that autophagy is activated in cardiac hypertrophy and ischaemia‐reperfusion injury.^10^, ^11^ Autophagy is related to the innate and adaptive immune systems,^12^, ^13^ and it is also closely associated with the NLRP3 inflammasome.^14^ Evidence suggests that autophagy can regulate macrophage activity and responses to stimuli,^15^, ^16^, ^17^ while the role of macrophage autophagy in MI is still unknown.

Calcium sensing receptor (CaSR), one of the seven‐transmembrane (7TM) receptors,^18^ works by sensing extracellular calcium concentration. CaSR is functionally expressed in the parathyroid, kidney and immune cells, including macrophages.^19^ Calhex231 is a specific inhibitor of CaSR^20^ which blocks Ca^2+^ by competitive binding to the 7TM domains.^21^ Calhex231 has been reported to exert a protective effect in cardiac hypertrophy^22^ and diabetic cardiomyopathy.^23^ Our previous study reported that CaSR activates the NLRP3 inflammasome by PLC‐IP3 in macrophages and amplifies inflammation and ventricular remodelling post MI^24^; however, the role and mechanism of Calhex231 in ventricular remodelling after MI remain unclear.

In this study, MI rats and peritoneal macrophages were treated with Calhex231, CaSR agonist, autophagy agonist, and antagonist or the inhibitor of NLRP3 inflammasome in order to elucidate the role and molecular mechanism of Calhex231 in myocardial fibrosis post MI.

## 2 MATERIALS AND METHODS

### 2.1 Chemicals

Calhex231, calindol hydrochloride (calindol), 3‐Methyladenine (3‐MA) and rapamycin (Rapa) were purchased from Sigma‐Aldrich (St. Louis, MO, USA). Z‐YVAD‐FMK was purchased from Enzo Life Science (Ave. Albert Einstein, Villeurbanne, FRA). Antibodies against NLRP3, LC3‐II/I, collagen I, pro‐/caspase‐1 (pro‐/casp‐1) and α‐sma were purchased from Abcam Inc (Cambridge, MA, USA). Antibodies against ASC, pro‐/IL‐1β and MMP9 were obtained from Santa Cruz Biotechnology (Santa Cruz, CA, USA). Antibodies against beclin‐1 and CaSR were obtained from Cell Signaling Technology (Danvers, MA) and Alomone Labs Ltd. (Hadassah Ein Kerem, Jerusalem), respectively. GAPDH antibody and all secondary antibodies were acquired from ZSGB‐Bio (Beijing, China). An IL‐1β ELISA kit was purchased from RD Systems (Minneapolis, MN, USA). Other chemicals and reagents used were all of analytical grade.

### 2.2 Rat MI model

Male Wistar rats (200 ± 20 g) were provided by the Animal Research Institute of Harbin Medical University. The experimental procedure was approved by the Institutional Animal Care and Use Committee of Harbin Medical University. All rats were raised in a 12h light/dark cycle and fed standard chow and water. After 1 week of adaptation, rats received a permanent ligation of the left anterior descending (LAD) artery.^25^ Briefly, rats were intubated and ventilated after being anaesthetized with 10% chloral hydrate (0.3 mL/100 g). Then, the heart was visualized via left thoracotomy, and the LAD was exposed and ligated under the left atrium by using the 4‐0 silk suture after separating the pericardium. Rats that died during anaesthesia resuscitation were not included in the analysis. After the operation, the rats were randomly divided into groups. The sham group: rats underwent the same procedure excluding LAD ligation (3 d, 7 d and 14 d, n = 30); the MI group: rats underwent the LAD ligation and had the same volume of vehicle injected (ip, once daily for 3 d, 7 d and 14 d, n = 45); and MI + Calhex231 group: MI rats were injected with Calhex231 (10 μmol/kg, ip, once daily for 3 d, 7 d and 14 d, n = 45). At the predetermined intervals (days 3, 7 and 14), rats were euthanized, and their hearts and peritoneal macrophages were collected after echocardiography had been performed. The hearts were stored at −80°C for subsequent investigation.

### 2.3 Isolation of rat peritoneal macrophages and groups

To identify the relationship between CaSR, autophagy, and the NLRP3 inflammasome in macrophages after MI, peritoneal macrophages were collected from the abdominal cavity of rats in each group by cold PBS aseptic lavage after anaesthesia. To detect the expression of the proteins, the cells were collected and prepared for Western blotting. Some peritoneal macrophages from MI 7 d rats were plated into 60 mm culture dishes containing 3 mL RPMI 1640.

To detect the relationship between CaSR and autophagy, cells were randomly divided into 3 groups: (a) the MI group; (b) the MI + calindol group: macrophages were incubated with 5 μmol/L calindol (CaSR activator) for 24 h; and (c) the MI + Calhex231 group: macrophages were preincubated with 5 μmol/L Calhex231 for 30 min, then calindol was added. To detect the relationship between autophagy and the NLRP3 inflammasome, macrophages were randomly divided into 4 groups: (a) the MI group; (b) the MI + Rapa group: macrophages were treated with 50 nmol/L Rapa for 24 h; (c) the MI + 3‐MA group: macrophages were pretreated with 5 μmol/L 3‐MA (a specific autophagy antagonist) for 30 min before Rapa was added; and (d) the MI + Z‐YVAD‐FMK group: macrophages were preincubated with 1 μmol/L Z‐YVAD‐FMK (a specific caspase‐1 inhibitor) for 30 min, then Rapa was added. After 24 h, supernatants and cell lysates were collected for ELISA and Western blotting.

### 2.4 Echocardiography

To evaluate the cardiac morphometry and function of rats in each group, a Vivid 7 Dimension echocardiographic system (GE Healthcare, Waukesha, WI, USA) was used to conduct echocardiography at predetermined intervals after anaesthesia (10% chloral hydrate, 0.3 mL/100 g). Heart rate (HR), left ventricular end‐systolic dimension (LVESD), left ventricular posterior wall diameter (LVPWD), left ventricular ejection fraction (LVEF) and left ventricular fractional shortening (LVFS) were measured and analysed.

### 2.5 HE and Masson staining

Following immersion in 4% paraformaldehyde for 24 h, the myocardial specimens were embedded in paraffin and cut into 4 μm sections. Then, the cardiac slices were stained with haematoxylin, eosin and Masson's trichrome reagent. These stained slices were viewed by light microscopy (Olympus BX60, Beijing, China). The collagen area was calculated by randomly selecting 10 visual fields (×400 magnification) from each cardiac section. The area of blue‐dyed collagen fibres, indicating fibrosis, was calculated using Image‐pro Plus software. The degree of fibrosis was determined by the ratio of the fibrotic area to the myocardial area.

### 2.6 Immunohistochemistry

To assess the infiltration of macrophages, CaSR, beclin‐1, LC3‐II/I, Casp‐1 and IL‐1β, immunohistochemical staining of cardiac slices was performed by microwave‐based antigen retrieval method.^26^ Antibodies against CD68 (1:100), CaSR (1:100), beclin‐1 (1:100), LC3‐II/I (1:100), Casp‐1 (1:100) and IL‐1β (1:100) were used. Under the microscope (Zeiss, Germany), cells with brown‐strained particles were deemed positive cells. Image‐Pro Plus software was used for quantitative analysis.

### 2.7 Immunofluorescence

Immunofluorescence was used to detect the expression of CaSR, autophagy, NLRP3 inflammasome and IL‐1β in myocardial infiltrating macrophages post MI. After antigen retrieval, antibodies against CD68 (1:100), CaSR (1:100), beclin‐1 (1:100), LC3II/I (1:100), NLRP3 (1:100), ASC (1:100), pro‐/Casp‐1 (1:100) and IL‐1β (1:100) were applied overnight at 4°C. The slices were respectively incubated with goat, mouse, or rabbit fluorescein isothiocyanate (FITC) or tetramethylrhodamine isothiocyanate (TRITC)‐conjugated secondary antibodies (1:100) for 2 h at 37°C. The slices were counterstained with DAPI (1:20,000) and covered with coverslip. A nonspecific IgGs stained slice was used as a negative control in parallel. Image Pro‐plus software was used to generate and analyse the colour composite images after images were acquired by IX70 microscope and MagnaFire 1.1 software at × 400 magnification.

### 2.8 Western blotting

After treatment, peritoneal macrophages were collected and proteins were extracted with a lysis buffer. Protein concentrations were measured with a BCA Protein Assay Kit (Bio‐Rad). After electrophoresis in 10% SDS‐PAGE, the protein was transferred to a polyvinylidene difluoride (PVDF) membrane. Before overnight incubation with the primary antibodies at 4°C, the membranes were blocked at room temperature for 2 h in 5% PBS‐dissolved nonfat dry milk. The primary antibodies used in the experiment were as follows: CaSR (1:800), beclin‐1 (1:1000), LC3 (1:1000), NLRP3 (1:1000), ASC (1:1000), pro‐/Casp‐1 (1:1000), pro‐/IL‐1β (1:1000), α‐sma (1:1000), collagen‐1 (1:1000), MMP9 (1:1000) and GAPDH (1:1000). Alkaline phosphatase‐conjugated secondary antibodies (1:5000) were used to incubate the membranes at room temperature for 1 h. Protein bands were obtained by Bio‐Rad ChemiDoc TM EQ densitometer, and the quantifications were measured with Bio‐Rad Quantity One software (Bio‐Rad, Hercules, CA, USA). GAPDH was used to normalize the ratio for the proteins examined.

### 2.9 IL‐1β concentration measurement

The concentration of IL‐1β in cell supernatant was detected using ELISA kit according to the manufacturer's instructions.

### 2.10 Statistical analysis

The data were collected from at least three independent experiments. Data are presented as the mean ± standard error of the mean (SEM). For normally distributed data, *t* tests were used to test the difference between two groups, and one‐way analysis of variance (ANOVA) was used between multiple groups, followed by Tukey's test. For non‐normally distributed data, the differences between two groups were evaluated by Mann‐Whitney U test, and Kruskal‐Wallis test was used between multiple groups. A *P*‐value of < 0.05 was considered to be statistically significant.

## 3 RESULTS

### 3.1 Calhex231 improved cardiac function and inhibited myocardial fibrosis post MI in rats

Echocardiographic evaluation of cardiac dimensions revealed that compared to the sham operation, MI significantly enlarged LVEDD and LVESD and led to dramatic decreases in LVEF and LVFS, which were markedly reversed by Calhex231 treatment. Concurrently, we found that the LVEF of MI rats was the worst at 7 d, and Calhex231‐mediated improvement was optimized at 7 d. HR did not change significantly among groups at all time points. LVPWD was not significantly different between the sham and MI groups until MI 14 d, but Calhex231 treatment did not significantly attenuate LVPWD (Table 1).

**TABLE 1:** Echocardiographic analysis of rats cardiac dimensions and left ventricular systolic function

		HR(bpm)	LVEDD(mm)	LVESD(mm)	LVPWD(mm)	LVEF(%)	LVFS(%)
	sham	466.5 ± 11.41	6.48 ± 0.30	3.81 ± 0.37	1.01 ± 0.02	77.2 ± 4.0	41.2 ± 3.3
3d	MI	426.0 ± 19.63	7.88 ± 0.14*	6.06 ± 0.15**	0.96 ± 0.40	36.5 ± 0.8***	15.1 ± 0.4***
	MI + Calhex231	395.5 ± 19.25	7.06 ± 0.28^#^	4.95 ± 0.31^#^	0.94 ± 0.49	51.7 ± 2.8^##^	23.0 ± 1.6^##^
	sham	471.3 ± 14.62	6.31 ± 0.36	3.77 ± 0.53	1.00 ± 0.02	76.1 ± 5.4	40.3 ± 4.4
7d	MI	466.3 ± 18.48	8.56 ± 0.26**	7.59 ± 0.26**	0.94 ± 0.077	27.9 ± 2.2**	11.3 ± 1.0**
	MI + Calhex231	456.7 ± 20.43	7.01 ± 0.43^#^	5.57 ± 0.50^#^	0.95 ± 0.07	47.3 ± 5.7^#^	20.6 ± 3.1^#^
	sham	474.0 ± 12.17	6.29 ± 0.34	3.47 ± 0.22	1.0 ± 0.02	81.1 ± 0.5	44.5 ± 0.4
14d	MI	458.3 ± 17.95	9.29 ± 0.51**	8.07 ± 0.43***	0.87 ± 0.02*	32.1 ± 0.4***	13.2 ± 0.2***
	MI + Calhex231	447.7 ± 13.17	7.74 ± 0.21^#^	6.32 ± 0.42^#^	0.94 ± 0.05	50.7 ± 2.9^##^	22.6 ± 1.6^##^

Abbreviations: HR, heart rate, LVEDD, left ventricular end‐diastolic dimension, LVESD, left ventricular end‐systolic dimension, LVPWD, left ventricular posterior wall diameter, LVEF, left ventricular ejection fraction, LVFS, left ventricular fractional shortening.

* *P*＜0.05, **P＜0.01, ***P＜0.001: MI group versus the sham group at same time point, ^#^ *P*＜0.05, ^##^ *P*＜0.01: MI + Calhex231 group versus the MI group in same time point.

**FIGURE 1:**
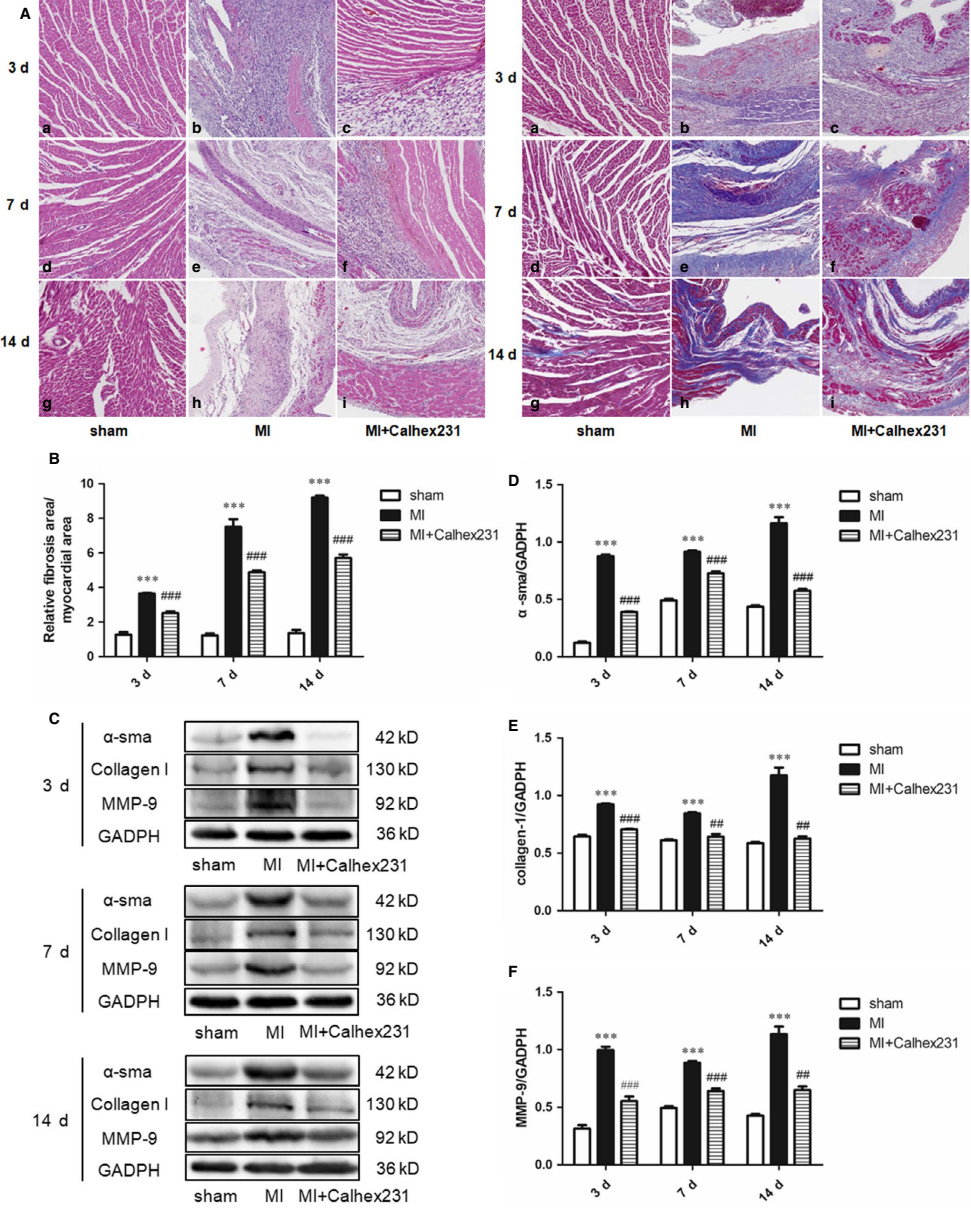
Calhex231 improved myocardial fibrosis post MI. (A) HE and Masson staining in the myocardium of sham and MI rats at each time point (×100). The MI group had obvious cardiomyocyte necrosis, accumulation of inflammatory cells, and scar tissue formation compared to sham group at same point in time, and Calhex231 ameliorated these changes. a‐i: HE staining, j‐r: Masson staining; n = 5. (B) Ratio of the fibrotic area to the myocardial area in the sham and MI rats at each time point. The ratio of the fibrotic area to the myocardial area was increased in the MI rats compared with the sham rats at each point in time, and Calhex231 attenuated this effect. ***P＜0.001 vs. sham group at the same time point, ^###^ *P*＜0.001 vs. MI group at the same time point. (C) Western blot analysis of α‐sma, collagen‐1 and MMP‐9 in the myocardium of sham and MI rats at each time point (n = 3). (D‐F) Bar graphs showing the quantitative analyses of the proteins α‐sma, collagen‐1 and MMP‐9. All of the proteins were up‐regulated in the MI rats compared to the sham rats, and they were down‐regulated by Calhex231. ***P＜0.001 vs. sham group at same time point, ^##^ *P*＜0.01, and ^###^ *P*＜0.001 vs. MI group at same time point

Compared to the sham group at same time interval, the MI group displayed obvious cardiomyocyte necrosis, accumulation of inflammatory cells and scar tissue formation detected by HE staining; Calhex231 ameliorated these changes (Figure 1 A, Figures S1 and S2). Moreover, Masson staining of the myocardium revealed that the ratio of fibrotic area to myocardial area was increased in slices of the myocardium in MI rats compared with similar parts in the sham group at each interval, and this effect was attenuated by Calhex231 (Figure 1 B). Proteins related to the myocardial fibrosis were detected by Western blot; the result showed that the expression of α‐sma, collagen I, and MMP‐9 was increased in the myocardium of the MI rats compared with that of sham rats and that they were decreased by Calhex231 (Figure 1 C‐F).

### 3.2 Calhex231 inhibited infiltration of inflammatory cells and activation of NLRP3 inflammasome post MI in rats

**FIGURE 2:**
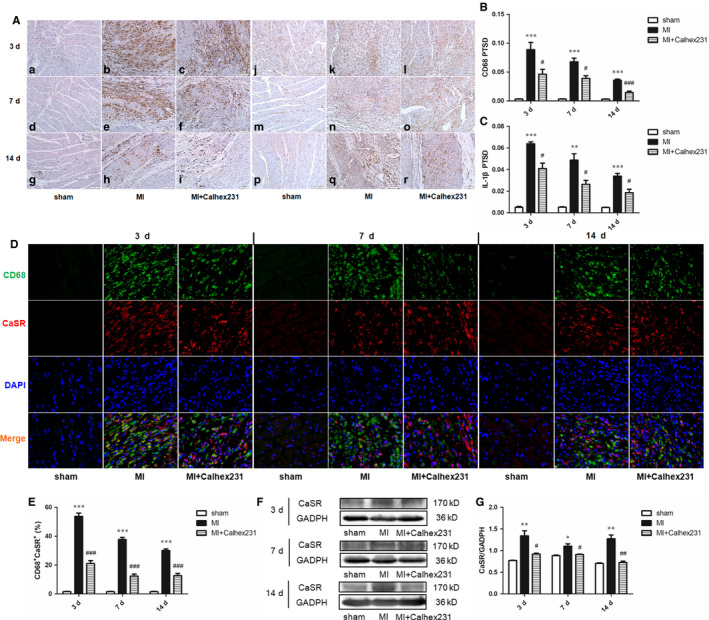
Calhex231 inhibited the infiltration of macrophages, IL‐1β and CaSR expression in rat myocardium and macrophages post MI. (A) Histochemical staining of CD68 and IL‐1β in the myocardium of sham and MI rats at each time point (×200). a‐i: histochemical staining of CD68, j‐r: histochemical staining of IL‐1β; (B, C) Quantification of the positive target surface density (PTSD) of the CD68^+^ and IL‐1β^+^ cells in cardiac tissues. The bar graphs show that CD68^+^ and IL‐1β^+^ cells were increased in the MI rats and decreased in the presence of Calhex231. Scale bar = 50 μm, n = 6. (D) Immunofluorescence staining of CD68^+^ and CaSR^+^ cells in the myocardium of sham and MI rats at each time point (×400). Immunofluorescence was used to detect the colocalization of CD68^+^ and CaSR^+^ cells in rat myocardium post MI. (E) Quantification of the double‐positive cells (%) in the cardiac tissues. The graphs show that CD68^+^ and CaSR^+^ cells were increased in MI rats and decreased in the presence of Calhex231 (n = 5). (F) Western blot analysis of CaSR in peritoneal macrophages at each time point. (G) Bar graphs showing the quantitative analyses of CaSR protein expression at different time points (n = 4). *P ＜ 0.05, **P ＜ 0.01 and ***P ＜ 0.001 vs. sham group at same time point. ^#^ *P *＜ 0.05, ^##^ *P *＜ 0.01 and ^###^ *P *＜ 0.001 vs. MI group at same time point

Histochemical findings showed that, compared with the levels in the sham rats, infiltration of macrophages (CD68^+^ cells) and IL‐1β (Figure 2A–C) were significantly increased in myocardial tissue after MI, while Calhex231 inhibited infiltration. Meanwhile, histochemical staining showed that at each interval, immunopositive cells of both CaSR and Casp‐1 were up‐regulated in the myocardium post MI, and Calhex231 reversed this effect (Figures S2 and S3 A, B).

**FIGURE 3:**
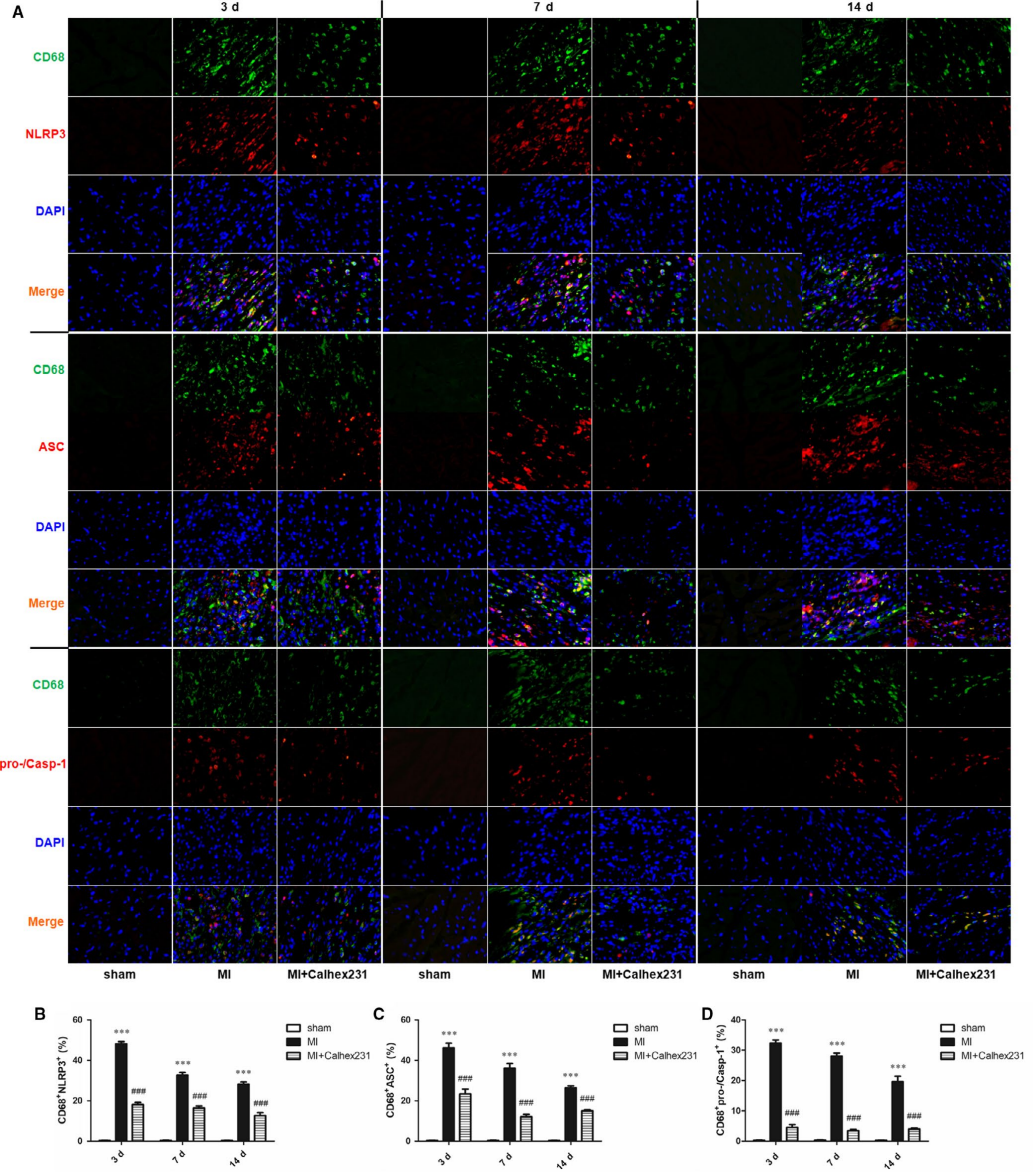
Calhex231 inhibited activation of the NLRP3 inflammasome in myocardial macrophages post MI. (A) Immunofluorescence staining of CD68^+^ and NLRP3^+^, ASC^+^, pro‐/Casp‐1^+^ cells in the myocardium of sham and MI rats at each time point (×400). Immunofluorescence was used to detect the colocalization of CD68^+^ and NLRP3^+^, ASC^+^ and pro‐/Casp‐1^+^ cells in the rat myocardium post MI. (B‐D) Quantification of the double‐positive cells (%) in the cardiac tissues. The graphs show that CD68^+^ NLRP3^+^, CD68^+^ ASC^+^ and CD68^+^ pro‐/Casp‐1^+^ were increased in the MI rats and decreased in the presence of Calhex231 (n = 5). *P ＜ 0.05, **P ＜ 0.01 and ***P ＜ 0.001 vs. sham group at same time point. ^#^ *P *＜ 0.05, ^##^ *P *＜ 0.01 and ^###^ *P *＜ 0.001 vs. MI group at same time point

**FIGURE 4:**
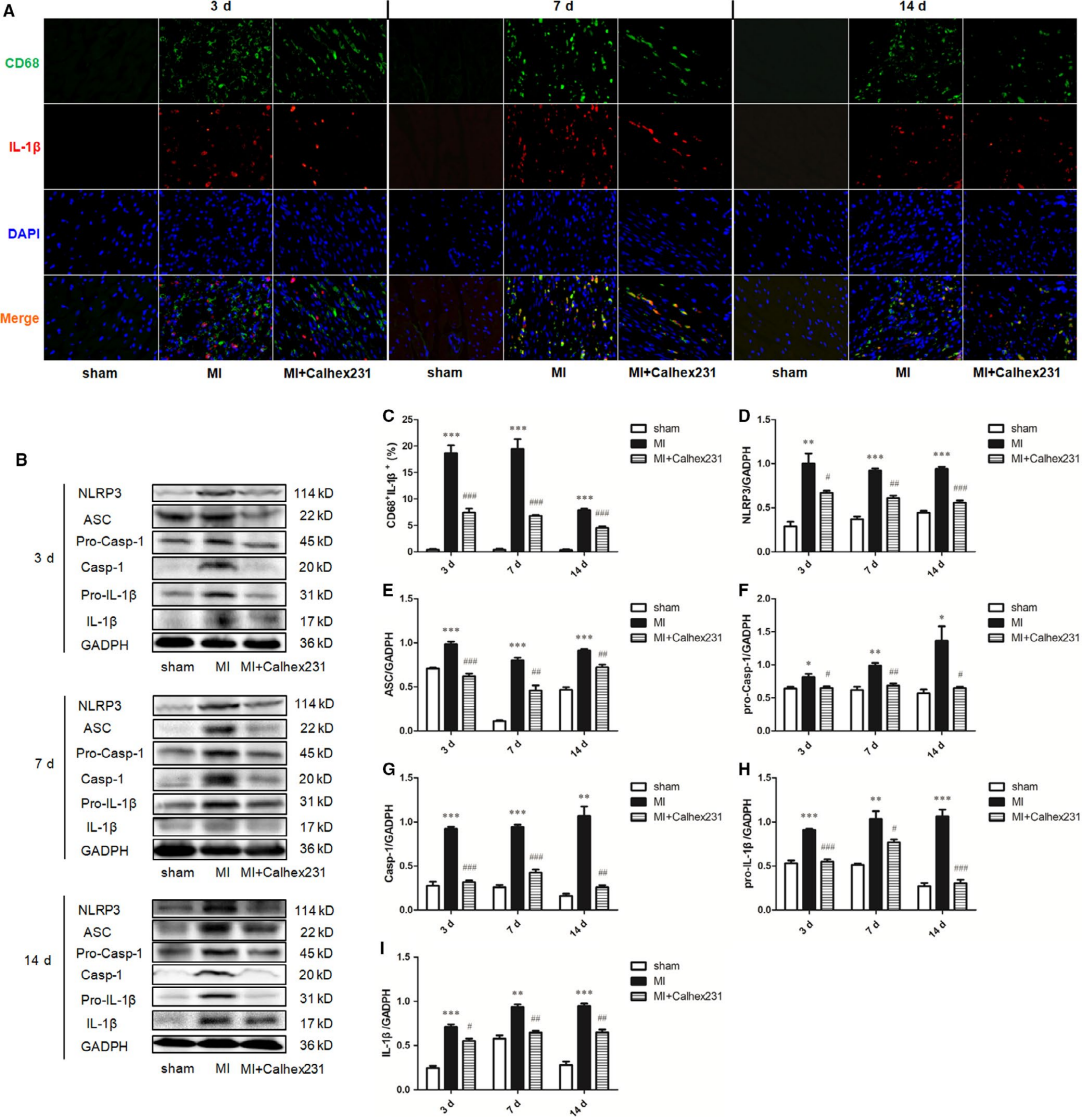
Calhex231 inhibited IL‐1β secretion in myocardial macrophages and activation of the NLRP3 inflammasome and IL‐1β secretion in peritoneal macrophages post MI. (A) Immunofluorescence staining of CD68^+^ and IL‐1β^+^ cells in the myocardium of sham and MI rats at each time point (×400). Immunofluorescence was used to detect the colocalization of CD68^+^ and IL‐1β^+^ cells in the rat myocardium post MI. (B) Western blot analysis of NLRP3, ASC, pro‐Casp‐1, Casp‐1, pro‐IL‐1β, and IL‐1β in peritoneal macrophages post MI at each time point. (C) Quantification of the double‐positive cells (%) in the cardiac tissues. The graphs show that CD68^+^ IL‐1β^+^ cells were increased in the MI rats and decreased in the presence of Calhex231 (n = 5). (D‐I) Bar graphs showing the quantitative analyses of the protein levels of NLRP3, ASC, pro‐Casp‐1, Casp‐1, pro‐IL‐1β and IL‐1β in peritoneal macrophages 3 d, 7 d, and 14 d post MI. n = 4. *P ＜ 0.05, **P ＜ 0.01, and *** *P *＜ 0.001 vs. sham group at same time point. ^#^ *P *＜ 0.05, ^##^ *P *＜ 0.01 and ^###^ *P *＜ 0.001 vs. MI group at same time point

Moreover, compared with that of the sham group rats at the same point in time, immunofluorescence analysis of myocardial tissue of the MI rats showed that the colocalization of CD68^+^ CaSR ^+^, CD68^+^ NLRP3^+^, CD68^+^ ASC^+^, CD68^+^ pro‐/Casp‐1^+^ and CD68^+^ IL‐1β^+^ cells was significantly increased, and the injection of Calhex231 significantly reduced the number of positive cells (Figures 2, 3, 4D,E, 3, 4A,C).

Additionally, the expressions of the CaSR, inflammasome component proteins NLRP3, ASC, and pro‐Casp‐1, activation marker Casp‐1, and effector molecule IL‐1β and its precursor pro‐IL‐1β were significantly increased in peritoneal macrophages in the MI group at each interval. However, Calhex231 inhibited the expression of these proteins (Figures 2F, G, and 4 B, D‐I).

### 3.3 Calhex231 reduced autophagy activation in rat myocardium and macrophages post MI

**FIGURE 5:**
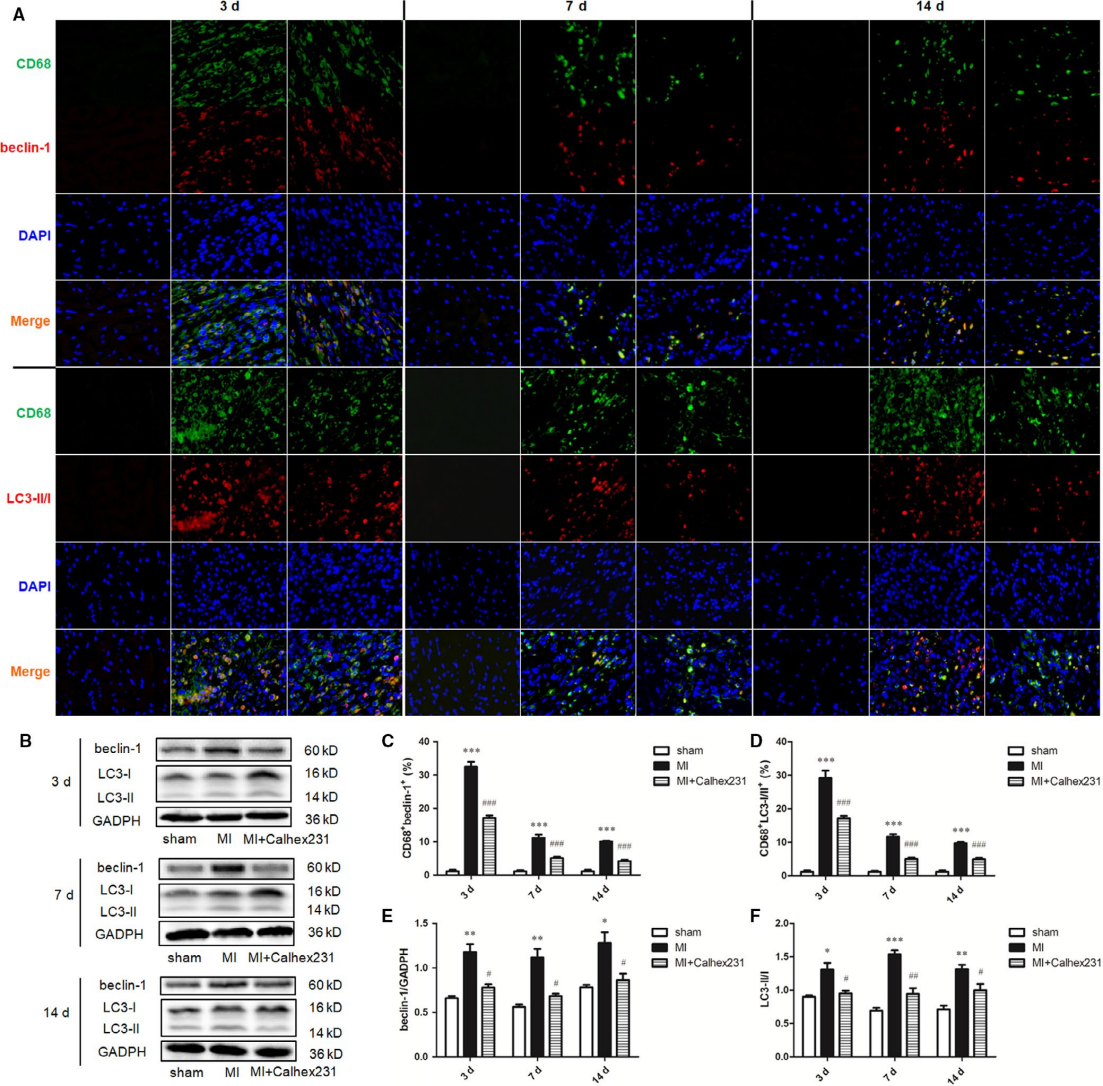
Calhex231 inhibited the expression of autophagy in macrophages after MI. (A) Immunofluorescence staining of beclin‐1^+^ and LC3‐II/I^+^ cells in the myocardium of sham and MI rats at each time point (×400). Immunofluorescence was used to detect the colocalization of CD68^+^ and beclin‐1^+^ and LC3‐II/I^+^ cells in the rat myocardium post MI. (B) Western blot analysis of beclin‐1 and LC3‐II/I in peritoneal macrophages at each time point. (C) Quantification of the double‐positive cells (%) in the cardiac tissues. The graphs show that CD68^+^ beclin‐1^+^ and CD68^+^ LC3‐II/I^+^ cells were increased in MI rats and decreased in the presence of Calhex231 (n = 5). (D‐F) Bar graphs showing the quantitative analyses of beclin‐1 and LC3‐II/I protein expression at 3 d, 7 d and 14 d (n = 6). *P ＜ 0.05, **P ＜ 0.01, and *** *P *＜ 0.001 vs. sham group at same time point. ^#^ *P *＜ 0.05, ^##^ *P *＜ 0.01 and ^###^ *P *＜ 0.001 vs. MI group at same time point.

By using histochemical staining, we observed increased levels of both beclin‐1 and LC3‐II immunopositive cells in the myocardium of the MI rats when compared with the sham rats at each interval, and Calhex231 decreased those levels (Fig. S 4 C‐F). The colocalization of beclin‐1 (CD68^+^ beclin‐1^+^) and LC3‐II/I (CD68^+^ LC3‐II/I^+^) on myocardium‐infiltrating macrophages was assayed by double‐dye immunofluorescence, and the result demonstrated that the colocalization of CD68^+^ beclin‐1^+^ and CD68^+^ LC3‐II/I^+^ cells was significantly increased in the MI rats at all points in time compared with the sham group, while Calhex231 reduced their colocalization (Figure 5A,C).

We also assessed the autophagy levels in the peritoneal macrophages that were extracted at each interval from the specimens. The expressions of beclin‐1 and LC3‐II/I in peritoneal macrophages were significantly increased in the MI rats compared with the sham rats, and the CaSR inhibitor Calhex231 significantly inhibited the expressions of these proteins at each point in time (Figure 5 B, D–F).

### 3.4 Calhex231 alleviated activation of autophagy and the NLRP3 inflammasome in peritoneal macrophages post MI in vitro

**FIGURE 6:**
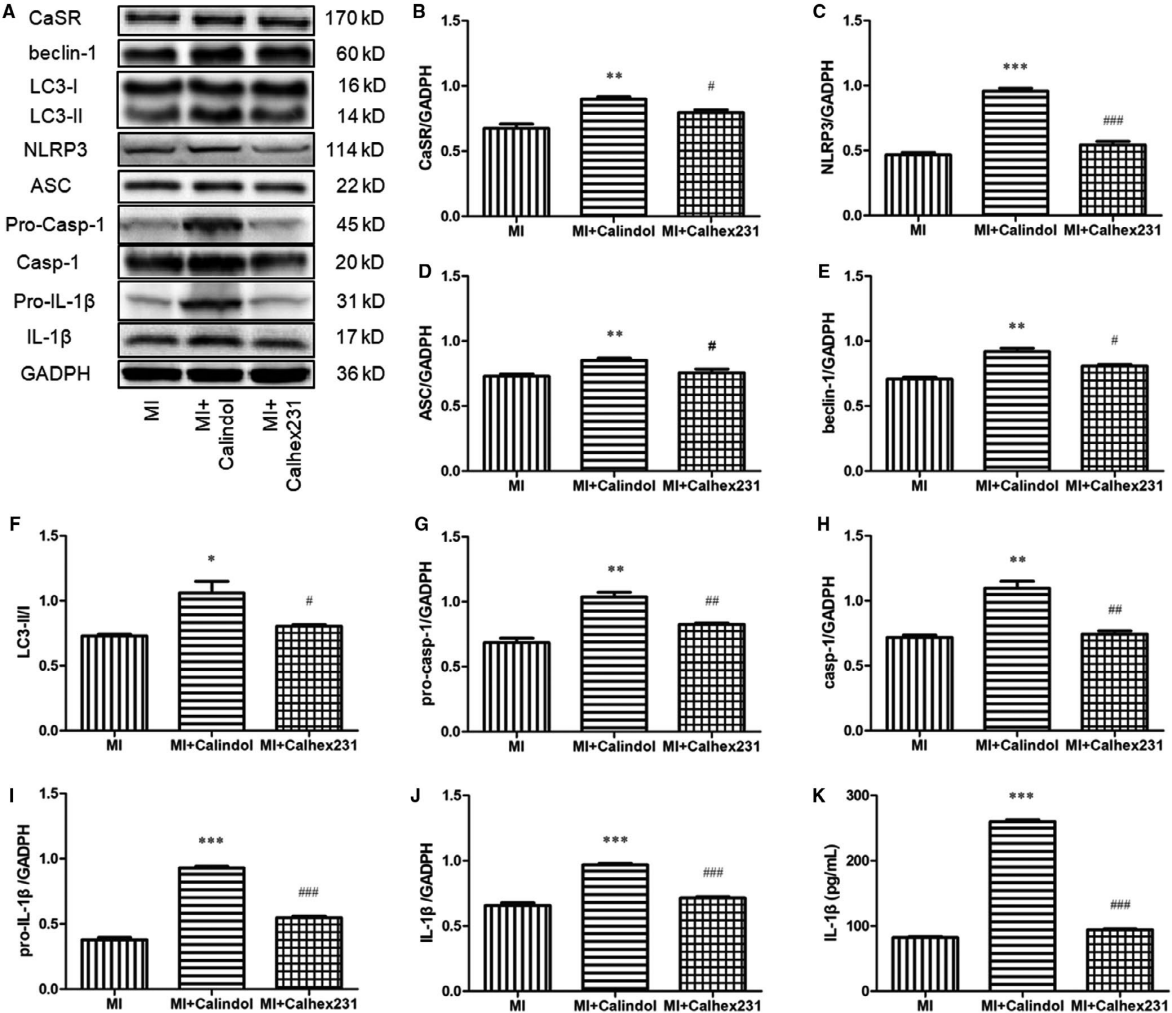
Calhex231 prevented activation of autophagy and the NLRP3 inflammasome in MI peritoneal macrophages in vitro. (A) Western blot analysis of CaSR, autophagy‐related proteins, components of the NLRP3 inflammasome, and IL‐1β expression. Peritoneal macrophages were extracted and activated or inhibited using calindol and Calhex231, and after 24 h treatment, the cell lyses were collected to detect the expression of CaSR, autophagy‐related proteins, components of the NLRP3 inflammasome and IL‐1β by Western blot. (B‐J) Quantitative analysis of the proteins from A (n = 4). (K) IL‐1β levels were measured by ELISA. *P ＜ 0.05, **P ＜ 0.01 and ***P ＜ 0.001 vs. MI group. ^#^ *P *＜ 0.05, ^##^ *P *＜ 0.01 and ^###^ *P *＜ 0.001 vs. MI+ Calindol group

Calindol, an agonist of CaSR, was used to detect the relationship between CaSR and autophagy in MI. Compared with the MI group, Calindol significantly increased the expression of CaSR, autophagy‐related molecules (beclin‐1 and LC3‐II/I), components of NLRP3 inflammasome (NLRP3, ASC and pro‐Casp‐1), the activated molecule of NLRP3 inflammasome (Casp‐1), and the effector of NLRP3 inflammasome (IL‐1β) and its precursor pro‐IL‐1β. These were significantly reversed by the application of Calhex231 (Figure 6A‐J). The level of IL‐1β detected by ELISA assay was consistent with that obtained by Western blot (Figure 6 K).

### 3.5 Autophagy was involved in NLRP3 inflammasome activation in peritoneal macrophages post MI in vitro

**FIGURE 7:**
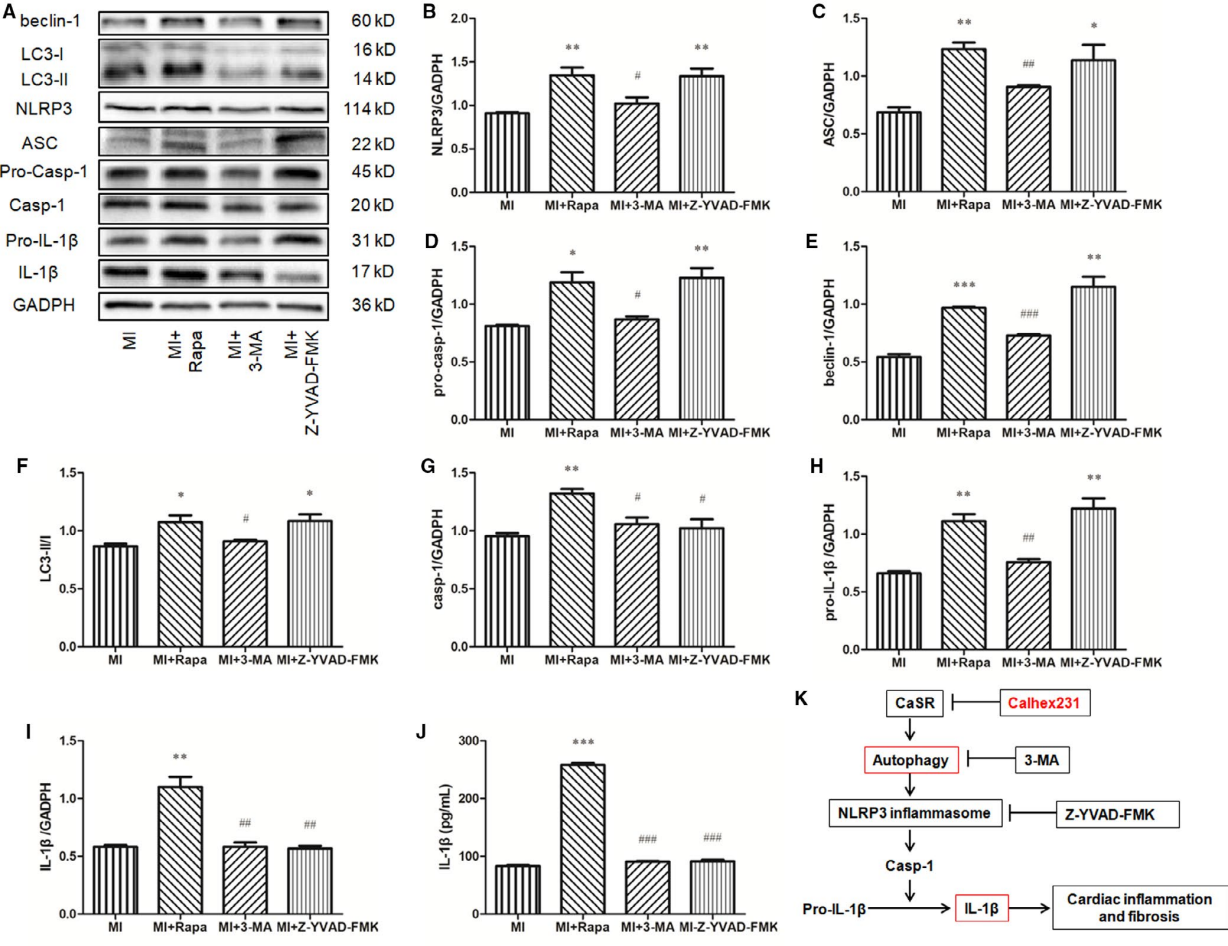
Autophagy was involved in NLRP3 inflammasome activation in MI peritoneal macrophages in vitro. (A) Western blot analysis of autophagy‐related proteins, components of the NLRP3 inflammasome, and IL‐1β expression in MI peritoneal macrophages. Peritoneal macrophages were extracted and activated or inhibited using Rapa and 3‐MA and Z‐YVAD‐FMK, and after 24 h treatment, the cell lyses were collected to detect the expression of CaSR, autophagy‐related proteins, components of the NLRP3 inflammasome and IL‐1β by Western blot. (B‐I) Quantitative analysis of the proteins from A. n = 4. (J) IL‐1β levels were measured with ELISA. *P ＜ 0.05, **P ＜ 0.01, and ***P ＜ 0.001 vs. MI group; ^#^ *P *＜ 0.05, ^##^ *P *＜ 0.01 and ^###^ *P *＜ 0.001 vs. MI + Rapa group. (K) A schematic diagram of the mechanism of the CaSR/autophagy/NLRP3 inflammasome in cardiac inflammation and fibrosis. In macrophages, an increase in autophagy induced by CaSR triggers NLRP3 inflammasome activation; the NLRP3 inflammasome induces IL‐1β, release, which leads to cardiac inflammation and fibrosis

Rapa (an autophagy agonist), 3‐MA (an autophagy specific inhibitor) and Z‐YVAD‐FMK (a specific inhibitor of caspase‐1) were used to elucidate the relationship of autophagy and the NLRP3 inflammasome. In peritoneal macrophages post MI, Rapa induced significant expression of beclin‐1, LC3‐II/I, NLRP3, ASC, pro‐Casp‐1, Casp‐1, pro‐IL‐1β and IL‐1β; conversely, 3‐MA inhibited their expression. Z‐YVAD‐FMK did not decrease the expression of beclin‐1, LC3‐II/I, NLRP3, ASC, pro‐Casp‐1 and pro‐IL‐1β, although it inhibited the level of Casp‐1 and IL‐1β (Figure 7 A‐I). Meanwhile, the ELISA detection of IL‐1β verified the results of Western blot (Figure 7J).

## 4 DISCUSSION

The results of this study show that Calhex231 ameliorated cardiac function and inhibited myocardial fibrosis, and infiltration of inflammatory cells post MI in rats. These protective effects of Calhex231 may be mediated through the inhibition of autophagy‐NLRP3 inflammasome pathway activation in macrophages post MI.

Ventricular remodelling has an important role in the development of heart failure after MI,^27^ mainly manifested as cardiac enlargement, myocardial fibrosis and cardiac dysfunction.^28^ In this study, echocardiographic indictors of cardiac dimensions and function, myocardial histological staining including HE staining and Masson staining, and myocardial fibrosis proteins markers in rats suggested that ventricular remodelling occurred after MI in rats, while the use of Calhex231, a specific CaSR inhibitor, could inhibit ventricular remodelling post MI in rats. These suggest that CaSR can be involved in the process of ventricular remodelling after MI.

Many studies have indicated that CaSR is involved in cell apoptosis, proliferation and inflammation.^29^, ^30^, ^31^ Additionally, CaSR participates in the pathophysiological processes of MI. Zhang et al reported that there was a positive correlation between myocardial CaSR expression and the degree of myocardial ischaemia/reperfusion injury in rats.^32^ Guo et al also reported that CaSR expression was positively correlated with the sensitivity of cardiomyocytes to MI in rats.^33^ In the present study, we successfully established a rat MI model as shown by significantly enlarged LVEDD and LVESD, dramatic decreases in LVEF and LVFS, obvious myocardial fibrosis, inflammatory cell accumulation and scar tissue formation compared to sham rats, which was consistent with the previous study.^25^ We also found that Calhex231 improved LV function, diminished the increased cardiac chamber size and reduced myocardial fibrosis induced by MI.

Calhex231, a member of allosteric calcilytics,^34^ can negatively modulate CaSR by binding to the extracellular part of the seven‐transmembrane domain of CaSR,^21^ which critically depends on residue E837 located in the seven‐transmembrane domain.^35^ Further study indicated the binding of Calhex231 is fixed on a salt bridge from the protonated secondary amino group on the ligands to the E837 residue.^36^ Our results suggested that after MI, CaSR was activated and up‐regulated, and the cardiac function, autophagy, inflammatory cell infiltration, inflammatory cytokine secretion and myocardial fibrosis of rats were aggravated. Many studies have shown that the concentration of Ca^2+^ was significantly increased in the infarct site after MI. In the presence of type I activators of CaSR such as Ca^2+^ and Mg^2+^, Calhex231 inhibits the activation of CaSR induced by Ca^2+^ through binding to the 7TM domain of CaSR which is far away from the Ca^2+^ orthosteric binding site.^37^ These results indicate that Calhex231 is a potential therapeutic drug for ventricular remodelling post MI, which suggests the pivotal role that CaSR plays in this process.

In this study, we found treatment with Calhex231 decreased infiltration of inflammatory cells identified by HE staining and CD68^+^ macrophages and IL‐1β shown by immunohistochemical staining in rat myocardium post MI. Our findings also demonstrate that Calhex231 may hinder ventricular fibrosis by inhibiting myocardial inflammation induced by macrophages and IL‐1β. Inflammation is involved in the initiation and progression of myocardial fibrosis.^38^

Monocytes are closely related to infarction size and LVEF in MI patients,^39^ and macrophages affect inflammation and heart failure post MI.^40^, ^41^ We reported that CaSR in macrophages increases ventricular remodelling by promoting the activation of the NLRP3 inflammasome.^24^ The NLRP3 inflammasome, which consists of NLRP3, ASC and pro‐Casp‐1, promotes pro‐Casp‐1 conversion into its activated form, Casp‐1,^8^ and triggers the activation of proinflammatory cytokines such as pro‐IL‐1β to IL‐1β; this results in initiation and amplification of inflammation‐mediated fibrotic processes.^42^ The NLRP3 inflammasome was examined in both myocardium‐infiltrating macrophages and peritoneal macrophages post MI to further investigate the influence of Calhex231 on its activation. All of the results demonstrated that Calhex231 diminished the expression of CaSR, the activation of NLRP3 components, activated Casp‐1, and IL‐1β and its precursor, both in myocardium‐infiltrating macrophages and peritoneal macrophages after MI. Artlett and Toldo indicated that the NLRP3 inflammasome directly regulates collagen synthesis, leading to collagen deposition in the lungs and heart,^43^ and that blocking Casp‐1‐activated IL‐1β improves ventricular remodelling after MI.^44^ The process of metabolism and proliferation in cardiomyocytes and cardiac fibroblasts is promoted by cytokines such as IL‐1β and TNF‐α, which are released by macrophages and T lymphocytes. These results suggest that Calhex231 can improve myocardial inflammation and fibrosis by suppressing NLRP3 inflammasome activity and subsequent IL‐1β release in macrophages post MI.

In the present study, we demonstrated that autophagy increased in both myocardium‐infiltrating macrophages and peritoneal macrophages after MI in rats but decreased in the presence of Calhex231. Calhex231 is a CaSR‐specific antagonist which plays an inhibitory role by blocking inositol phosphates and decreasing Ca^2+^ concentrations.^21^ Studies have shown that activation of autophagy is closely related to Ca^2+^.^45^ Calhex231 inhibits autophagy by inhibiting cardiomyocyte [Ca^2+^]_i_, thereby alleviating myocardial hypertrophy.^46^ Our results indicate that macrophage autophagy was activated in MI rats and might be inhibited by Calhex231. However, the precise regulative role of Calhex231 in cardiomyocyte [Ca^2+^]_i_ post MI requires further study. In this study, we found that the NLRP3 inflammasome and autophagy were activated post MI in vivo, and Calhex231 inhibited their activation. To further clarify their relationship, we conducted in vitro experiments on peritoneal macrophages from the MI rats. The results showed that calindol, which plays a positive regulating role by binding to the Ca^2+^ synergistic site on the 7TM of CaSR,^37^ activated autophagy in macrophages post MI. In contrast, Calhex231 pretreatment in vitro inhibited this process. These experimental results creatively showed that macrophage autophagy is activated and induced by CaSR post MI. It has been reported that macrophage autophagy is closely related to the NLRP3 inflammasome.^14^ Previous studies have shown that deficiency in macrophage autophagy strengthens NLRP3 inflammasome activation and silica exposure‐induced chronic lung disease,^47^ as well as promoting angiotensin II‐induced inflammation and cardiac fibrosis.^48^ Our study also showed that Rapa, an agonist of autophagy, increased MI‐induced autophagy, NLRP3 activation and IL‐1β secretion; however, specific antagonist of autophagy 3‐MA diminished these effects. Interestingly, Z‐YVAD‐FMK, which serves as a specific inhibitor of NLRP3 inflammasome and acts by inhibiting activation of caspase‐1, inhibited NLRP3 inflammasome activity and the release of IL‐1β, but not autophagy. These findings suggest that inhibiting autophagy can suppress activation of the NLRP3 inflammasome and subsequent IL‐1β release in peritoneal macrophages post MI, indicating that Calhex231 might have attenuated the NLRP3 inflammasome activity through inhibiting autophagy in macrophages post MI. The signalling pathway of autophagy that is suppressed by Calhex231 needs to be investigated further.

There were limitations in the study. First, rat peritoneal macrophages were extracted with intraperitoneal injection of cold PBS combined with abdominal massage for in vitro experiments. Although the role of macrophages in the development of ventricular remodelling post MI is partially explained in our in vitro study, macrophages infiltrated in myocardial tissue post MI were extracted by magnetic microbeads for in vitro experiments to better reflect the functional status of macrophages in the local myocardial tissues of MI. However, there is still no magnetic microbeads for rat macrophages at present. Second, we extracted peritoneal macrophages from MI rats for in vitro study, emphasizing the role of macrophages under the MI condition. Even so, the comparison of peritoneal macrophages from rats in the control group may reflect the effect of macrophages in different states on the process of ventricular remodelling after MI. These are worth further study.

In summary, we demonstrated that Calhex231 improved cardiac function, attenuated myocardial inflammation and fibrosis, and decreased activation of autophagy and the NLRP3 inflammasome in macrophages post MI. In contrast, treatment of macrophages with Calhex231 markedly attenuated autophagy‐mediated up‐regulation of the NLRP3 inflammasome. Our results suggest that Calhex231 ameliorates myocardial fibrosis post MI in rats, likely through inhibiting autophagy‐NLRP3 inflammasome pathway in macrophages. These may provide a new therapeutic target for ventricular remodelling‐related cardiovascular diseases, including MI, hypertension and heart failure.

## CONFLICTS OF INTEREST

The authors have no conflicts of interest to declare.

## AUTHOR CONTRIBUTIONS

Wenxiu Liu: designing research and writing the paper; Jiaxing Sun, Yutong Guo, Na Liu and Xue Ding: performing research; Xin Zhang, Jinyu Chi and Ningning Kang: analysing data and revising the manuscript; Yue Liu and Xinhua Yin: supervising the experiments, revising and approving the manuscript.

## Supporting information

Fig S1

Fig S2

Fig S3

Fig S4

Supplementary Material

## Data Availability

The experimental data may be obtained from the corresponding author upon reasonable request.
