# Supplementary figures and images for: Calhex231 ameliorates myocardial fibrosis post myocardial infarction in rats through the autophagy‐NLRP3 inflammasome pathway in macrophages
Source: J Cell Mol Med. 2020 Oct 12;24(22):13440–53. doi: 10.1111/jcmm.15969 (PMC7701583; doi:10.1111/jcmm.15969)

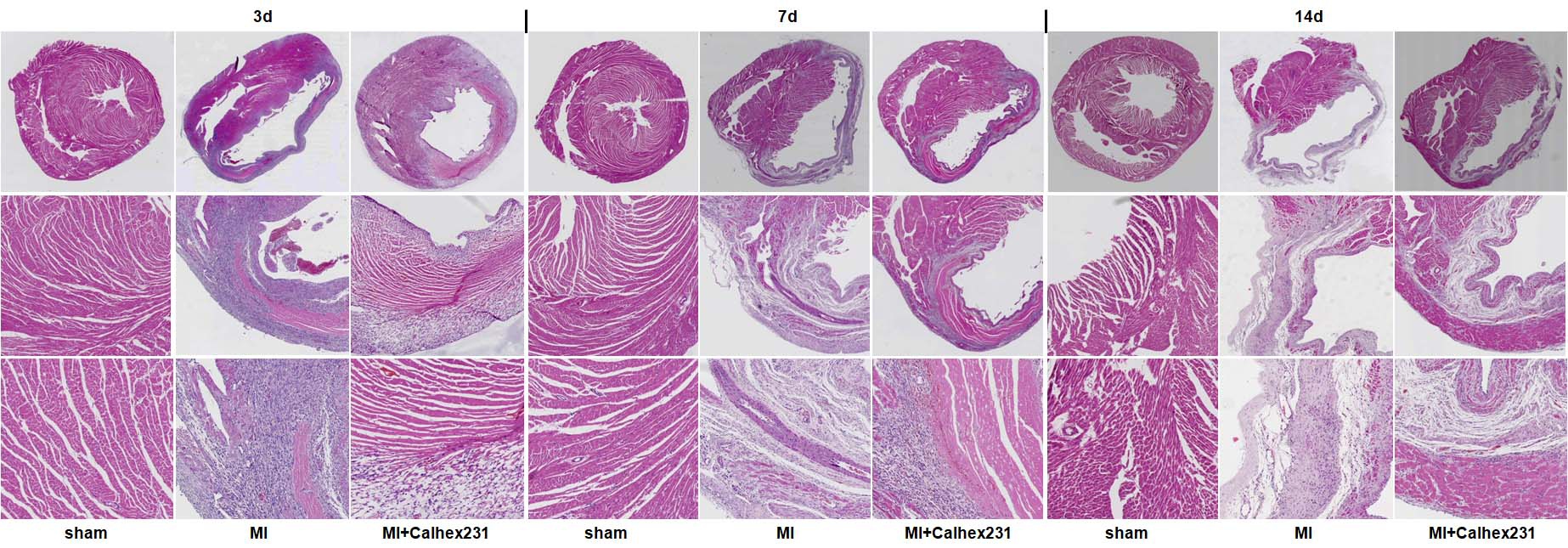

Supplement: Supplementary file 1 — Fig S1 [file JCMM-24-13440-s001.tif]

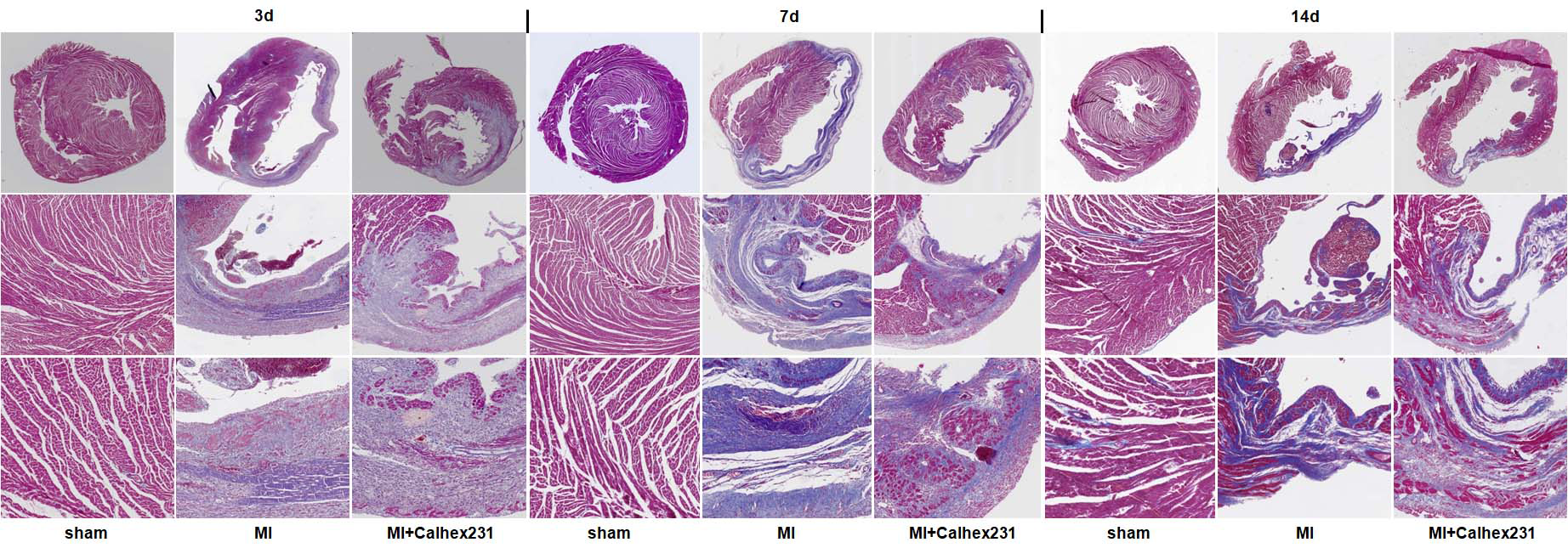

Supplement: Supplementary file 2 — Fig S2 [file JCMM-24-13440-s002.tif]

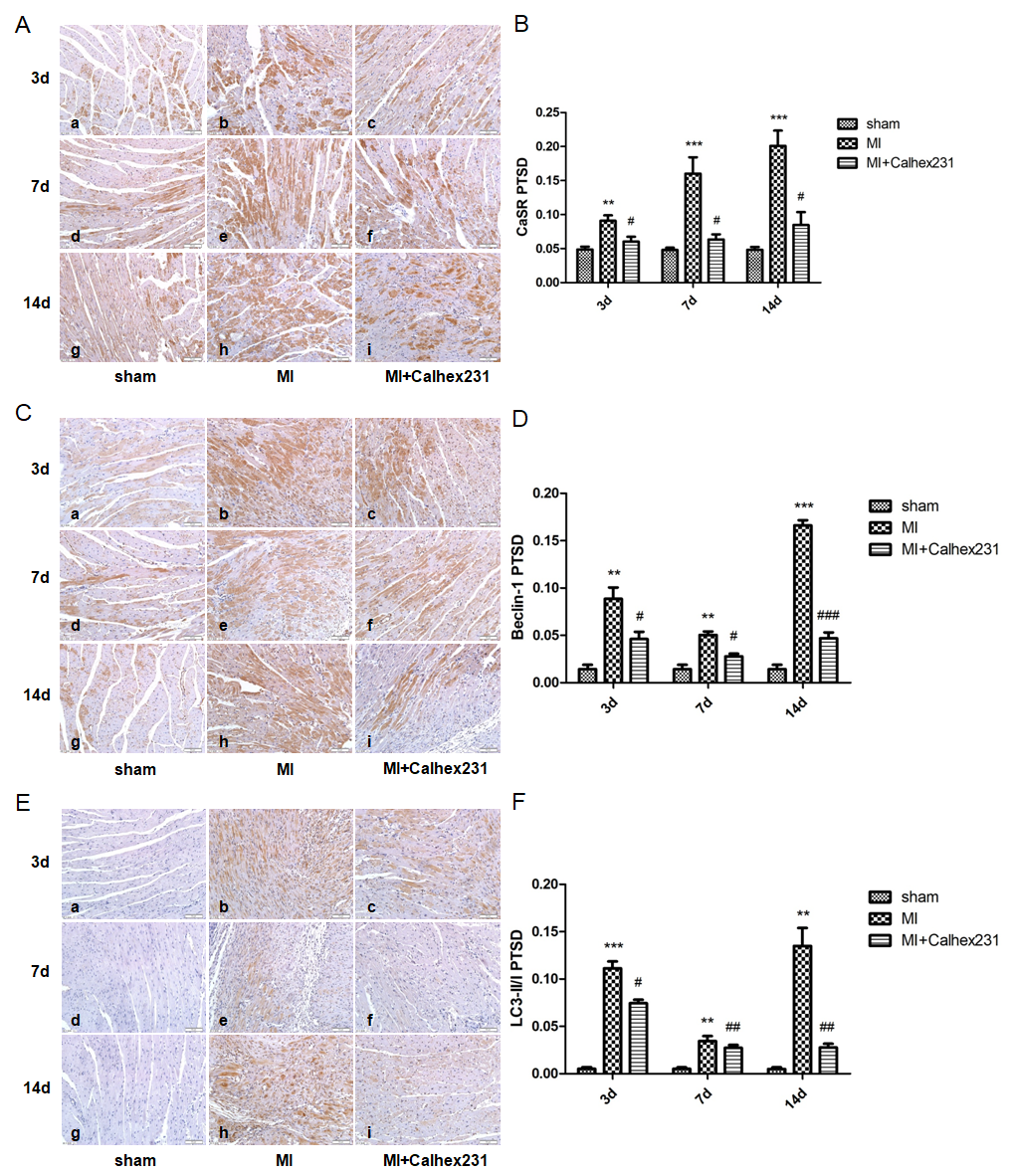

Supplement: Supplementary file 3 — Fig S3 [file JCMM-24-13440-s003.tif]

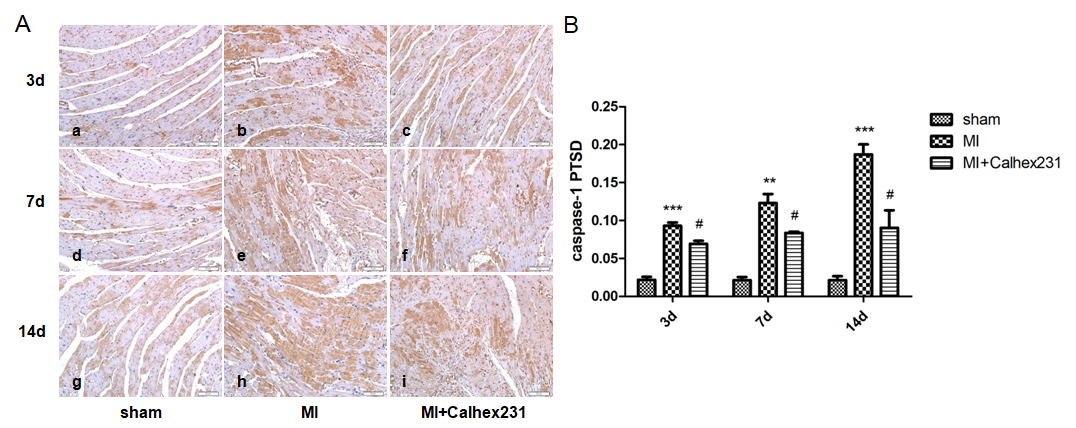

Supplement: Supplementary file 4 — Fig S4 [file JCMM-24-13440-s004.tif]
