# Supplementary material for: Calhex231 ameliorates myocardial fibrosis post myocardial infarction in rats through the autophagy‐NLRP3 inflammasome pathway in macrophages
Source: J Cell Mol Med. 2020 Oct 12;24(22):13440–53. doi: 10.1111/jcmm.15969 (PMC7701583; doi:10.1111/jcmm.15969)
Supplement: Supplementary file 5 — Supplementary Material [file JCMM-24-13440-s005.docx]

**Fig. S 1 Calhex231 ameliorated myocardial inflammation post MI.** HE staining of representative myocardial tissues of rats in different groups and time points at different magnifications (×10, ×40, ×100). The MI group had obvious cardiomyocyte necrosis, accumulation of inflammatory cells, and scar tissue formation compared to sham group at same point in time, and Calhex231 ameliorated these changes. n = 5.

**Fig. S 2 Calhex231 ameliorated myocardial fibrosis post MI.** Masson staining of representative myocardial tissues of rats in different groups and time points at different magnifications (×10, ×40, ×100). The MI group had obvious fibrosis compared to sham group at same point in time, and Calhex231 ameliorated these changes. n = 5.

**Fig. S 3 Calhex231 inhibited the expression of cardiac CaSR and autophagy in the myocardium post MI.** **(A)** Histochemical staining of CaSR, beclin-1 and LC3 in the sham and MI rat myocardiums at each time point (×200). Immunohistochemistry was used to detect the expression of CaSR, beclin-1 and LC3 in the cardiac tissue of rats. **(B)** Quantification of the positive target surface density (PTSD) of the CD68^+^ and IL-1β^+^ cells in the cardiac tissues. The graphs demonstrated that CaSR, beclin-1 and LC3 increased in MI rats and decreased in the presence of the of Calhex231. bar = 50 μm, n = 5. **P*＜0.05, ***P*＜0.01, and ****P*＜0.001 vs. sham group at same time point, ^#^*P*＜0.05, ^##^*P*＜0.01, and ^###^*P*＜0.001 vs. MI group at same time point.

**Fig. S 4 Inhibition of CaSR reduced cardiac NLRP3 inflammasome activation post MI**. **(A)** Histochemical staining of Casp-1 in the sham and MI rat myocardiums at each time point (×200). Immunohistochemistry was used to detect the expression of Casp-1 in the cardiac tissue of rats. **(B)** Quantification of the positive target surface density (PTSD) of the Casp-1^+^ cells in the cardiac tissues. The graphs demonstrated that Casp-1 increased in MI rats and decreased in the presence of Calhex231; bar = 50 μm, n = 6. **P*＜0.05, ***P*＜0.01, and ****P*＜0.001 vs. sham group at same time point. ^#^*P*＜0.05 vs. MI group at same time point.
